# The delivery of compassionate nursing care in a tick-box culture: Qualitative perspectives from a realist evaluation of intentional rounding

**DOI:** 10.1016/j.ijnurstu.2020.103580

**Published:** 2020-07

**Authors:** Sarah Sims, Mary Leamy, Ros Levenson, Sally Brearley, Fiona Ross, Ruth Harris

**Affiliations:** a Florence Nightingale Faculty of Nursing, Midwifery and Palliative Care, King's College London, London, UK; b Independent Researcher, London, UK; c Centre for Health and Social Care Research, Kingston University and St George's University of London, London, UK

## Abstract

**Background:**

Compassion is integral to professional nursing practice worldwide and a fundamental value in healthcare. Following serious care failures at a healthcare provider in the United Kingdom, a government commissioned report (the Francis Report) made several recommendations for strengthening compassion in nursing care and consequently ‘intentional rounding’ was incorporated into nursing practice in the United Kingdom. Intentional rounding is a structured process implemented primarily in the United Kingdom, North America and Australia, whereby nurses conduct 1–2 hourly checks on every patient using a standardised protocol and documentation.

**Objectives:**

To examine the role of intentional rounding in the delivery of compassionate nursing care in England from multiple perspectives.

**Methods:**

This paper reports qualitative findings from one phase of a realist evaluation of intentional rounding which used a mixed-methods approach. Individual, semi-structured interviews were undertaken with 33 nursing staff, 17 senior nurse managers, 34 patients and 28 family carers from three geographically spread case study hospital sites in England. Interviews elicited detailed reflections on the contexts, mechanisms and outcomes of intentional rounding and how it impacted the interviewee and those around them.

**Results:**

This study found little evidence that intentional rounding ensures the comfort, safety or dignity of patients or increases the delivery of compassionate care. The systematised approach of intentional rounding emphasises transactional care delivery in the utilisation of prescribed methods of recording or tick boxes rather than relational, individualised patient care. It has the potential to reduce the scope of nursing care to a minimum standard, leading to a focus on the fundamentals as well as the prevention of adverse events. Its documentation is primarily valued by nursing staff as a means of protecting themselves through written proof or ‘evidence’ of care delivered, rather than as a means of increasing compassionate care.

**Conclusions:**

This large-scale, theoretically-driven study of intentional rounding – the first of its kind – demonstrates that intentional rounding prioritises data collection through tick boxes or a prescriptive and structured recording of care. Thus, intentional rounding neither improves the delivery of compassionate nursing care nor addresses the policy imperative it was intended to target. This study raises questions about the role, contribution and outcomes from intentional rounding and suggests a need for a wider, international debate within the nursing profession about its future use. If an intervention to increase compassionate nursing care is required, it may be better to start afresh, rather than attempting to adapt the system currently implemented.

## What is already known about the topic?

• Global healthcare service provision is becoming increasingly complex in response to changes in population demographics, increasing health inequalities, advancing technology and imperatives for financial efficiency. Alongside these systems pressures, and regardless of them, compassion is, and will remain, a central tenet of nursing practice, integral to professional practice and a fundamental value of healthcare worldwide.
• Intentional rounding was incorporated into nursing practice across the UK as part of a swift policy reaction to care failures at Mid Staffordshire NHS Trust and in a bid to improve compassionate nursing care.
• There was an unclear implementation strategy for intentional rounding in England and substantial limitations to its evidence base.

## What this paper adds

• This study found little evidence that intentional rounding ensures the comfort, safety or dignity of patients or increases the delivery of compassionate nursing care.
• The systematised approach of intentional rounding emphasises transactional care delivery rather than relational, individualised patient care and has the potential to commodify nursing and reduce the scope of nursing care to a minimum standard.
• The majority of patients and family carers interviewed had not been aware that intentional rounding was occurring. They said they valued the relational elements of their interactions with nursing staff; the humour, responsiveness and friendliness of communications which made them feel cared for and reassured but such elements of communication are not a prominent feature in the intentional rounding protocol.
• The study concludes there is a need for an international debate within the nursing profession to consider what its desired outcomes are and whether intentional rounding is the best way of achieving them.

## 1 Introduction

The context of global healthcare service provision is becoming increasingly complex in response to key challenges and opportunities, such as changes in population demographics and social circumstances, advancing technology and financial efficiency (World Health Organisation, 2016). Alongside these changes, compassionate care remains central to nursing, integral to professional practice and a fundamental value of healthcare worldwide (e.g. International Council of Nursing, 2012; American Nursing Association, 2015; Nursing and Midwifery Council, 2008; 2010; Department of Health, 2010; 2012; Health Service Ombudsman, 2011). The American Nurses Association prioritises compassion as a non-negotiable ethical standard and states:

*‘The nurse practices with compassion and respect for the inherent dignity, worth, and unique attributes of every person.’* (American Nurses Association, 2015)

Similarly, the NHS Constitution states:

*‘Compassion…we respond with humanity and kindness to each person's pain, distress, anxiety or need. We search for the things we* can *do, however small, to give comfort and relieve suffering. We find time for those we serve and work alongside. We do not wait to be asked, because we care.’* (Department of Health, 2010: p239)

Many different definitions of compassion have been highlighted in the nursing literature (e.g. Schantz, 2007; Chambers and Ryder, 2009; Crawford et al., 2014; Sinclair et al., 2016). Due to its subjective nature, defining and understanding the true meaning of compassion remains elusive and complex (Straughair, 2012b; Van Der Cingel, 2009; 2011). Compassion has been varyingly referred to as a feeling (Goetz et al., 2010); an emotion or sentiment (Morse et al., 2006); an attitude (Sprecher and Fehr, 2005); an altruistic expression (Birch, 1993); and a moral virtue (Dietze and Orb, 2000). Evidence suggests that compassion requires a variety of skills, including noticing, engaging, connecting and showing humanity and humility (Goodrich and Cornwell, 2008; Dewar et al., 2014). To add further complexity, the term ‘compassion’ is also used interchangeably with ‘caring’, ‘empathy’, ‘kindness’ and ‘sympathy’ (Schantz, 2007; Bradshaw, 2009). Perhaps contestably, it has been argued that until compassion is clearly defined, nurses will not be able to truly claim it as an integral dimension of professional practice (Olshansky 2007; Straughair, 2012b). Not surprisingly, given the subjective and complex nature of compassion, its measurement is difficult. Compassion manifests differently in different contexts and means different things to different people (Dewar et al., 2011), therefore there is a danger of simply measuring what is easy to quantify in compassionate care, rather than what is important (Chochinov, 2007; MacLeod and McPherson, 2007).

Accounts of an erosion of compassionate nursing care or a ‘crisis of caring’, both worldwide and within the UK, are all matters of professional and public debate and are of increasing concern (Straughair, 2012a; 2012b; Scott, 2014; Parliamentary and Health Service Ombudsman, 2011; Youngson, 2008). Whilst in general, nurses across the NHS are striving to deliver high-quality care (Proctor 2008), unfortunately examples of poor nursing care, with deplorable lapses in human kindness and compassion, have been highlighted. In 2013, Sir Robert Francis’ landmark public inquiry, report and recommendations (Francis, 2013) highlighted accounts of poor nursing care and the lack of dignity afforded to patients at the Mid Staffordshire NHS Foundation Trust in England between 2005 and 2009. One of the concerns raised by the Francis Report was the neglect by some nursing staff to meet patients’ fundamental care needs, such as toileting, pain, hydration, comfort and safety. Reports were made of patients left in soiled bed linen for long periods of time; of a failure to provide food or to assist patients to eat; and of a lack of attention to patients’ personal hygiene (Francis, 2013). As a result, the report made several recommendations for improvement in a number of areas, including the education and training of nurses, monitoring of nursing performance and more effective management and leadership of nursing staff (Straughair, 2012a). A further recommendation, aimed at improving compassionate nursing care, was that:

“*Regular interaction and engagement between nurses and patients and those close to them should be systematised though regular ward rounds”* (Francis, 2013, Vol III, p1610).

This recommendation rapidly became synonymous with intentional rounding in professional discourse. Intentional rounding is a structured process developed in the US (Studer, 2007), which involves nurses carrying out one to two hourly checks of patients’ fundamental care needs using a standardised protocol and documentation (Box 1). It has since been implemented primarily throughout the UK, North America and Australia. It was primarily introduced in the US as a way for hospitals to improve patient experience scores required as part of funding eligibility (Blakley et al., 2011; Willis et al., 2016). In Australia, there are also reports of intentional rounding being implemented as a risk reduction strategy in response to missed care (Willis et al., 2016).

Box 1. Typical intentional rounding schedule in acute ward settings (Bartley, 2011; Studer, 2007)

During each round, the following standardised protocol is used by a nurse for each patient:

• an opening phrase is used by the nurse to introduce his or herself and to put the patient at ease;
• scheduled tasks are then performed;
• a discussion of the four key elements of the round, often called the ‘4 P's':

○ Positioning - making sure the patient is comfortable and assessing the risk of pressure sores;
○ Personal needs - assessing the patients’ personal needs, including whether they need assistance with getting to the toilet;
○ Pain - asking patients to rate their level of pain on a scale of 0–10;
○ Placement - ensuring any items a patient needs are within easy reach;

• an assessment of the care environment, such as checking the temperature of the room or any fall hazards;
• ending the interaction with a closing phrase such as “Is there anything else I can do for you before I go?”;
• the patient is informed of when the nurse will return;
• the nurse documents the round.

If patients are unable to respond during the round, the nurse may follow this process with family members (Halm, 2009).

*Box as cited in* *Harris et al. (2017)*

Alt-text: Unlabelled box

At this crisis point in the UK and in response to widespread public concern, the then Prime Minister of the UK, David Cameron, announced in January 2012 that all nurses should undertake hourly rounds to ensure the comfort, safety and dignity of their patients. Although not a statutory requirement or mandatory, this proposal was supported by the Chief Nursing Officer for England and therefore expected to happen at speed (Harris et al., 2019). A small number of Trusts had implemented intentional rounding prior to publication of the Francis report, but this was the main driver for the majority of Trusts. There was no consultation with stakeholders or the nursing profession or development of accompanying guidelines or resources to support the implementation of intentional rounding other than that it was to be delivered hourly by Registered Nurses (RNs) using the ‘4P's’ format (Box 1). Aware of the lack of implementation guidelines, the Nursing and Care Quality Forum (NCQF) supported the development of seven ‘demonstrator sites’ (i.e. early adopters of intentional rounding) and commissioned a small report to enable lesson learning for other Trusts (Levenson, 2013). Unfortunately, this report was published in August 2013, when widespread implementation of intentional rounding was already underway. Therefore, the moment to use the learning to inform system-wide implementation was missed. At this point, the role of the NCQF had become unclear and it is not known how widely this report was circulated. The report was published on an NHS website (www.6cs.england.nhs.uk) but is no longer publicly available.

In the light of the unclear implementation strategy for intentional rounding and the substantial limitations to its evidence base including poor quality studies and the misreporting of findings (National Nursing Research Unit, 2012; Snelling, 2013a; 2013b), a study was funded by the National Institute for Health Research, Health Services and Delivery Research programme (NIHR HS&DR) in 2014 to investigate the impact of intentional rounding in hospital wards on the organisation, delivery and experience of care (Harris et al., 2019). This was the first theoretically-driven, large-scale, mixed-methods evaluation of intentional rounding to be conducted worldwide. A realist evaluation (Pawson and Tilley, 1997) of intentional rounding was undertaken using a multi-method study design. A theory-driven approach designed for evaluating complex social interventions, realist evaluation acknowledges that complex social interventions only ever work for certain people in particular circumstances (Hewitt et al., 2012; Pawson et al., 2004). Therefore, instead of asking the question, ‘Does this intervention work?’ it asks, ‘What is it about this intervention that works, for whom and in what circumstances?’ The study was conducted in four phases. This paper reports the findings of the qualitative interviews conducted with staff, patients and family carers in Phase 3 of the study only.

Phase 1 of the study involved theory development using realist synthesis (Pawson et al., 2004, 2005) of the international literature to generate hypotheses about what the mechanisms of intentional rounding may be, what particular groups may benefit most or least and what contextual factors might be important to its success or failure. These programme theories provide the theoretical basis for the study and were used as a framework to test emerging findings from subsequent research phases. Phase 2 involved a national survey of all NHS acute Trusts (an organisation that provides hospital-based services within the English National Health Service) to explore how intentional rounding was implemented and supported across England. Phase 3 involved in-depth case studies of six wards involving individual interviews, observations, retrieval of routinely collected ward outcome data and an analysis of costs. The methods from the individual interviews component of Phase 3 are reported in more detail in the Methods section of this paper. Phase 4 involved analysing data accumulated across the phases for patterns of congruence and discordance and to develop an overall evaluation of what aspects of intentional rounding work, for whom and in what circumstances.

More detailed information on the realist synthesis (Sims et al., 2018), the study methods for the full realist evaluation (Harris et al., 2017) and the whole study findings (Harris et al., 2019) have been published elsewhere. This paper focuses on one aspect of the findings from Phase 3 of the mixed methods study – the qualitative interviews with hospital staff, patients and family carers. This paper expands upon the qualitative interview findings reported in Harris et al. (2019), presenting additional and more detailed analysis of the role of intentional rounding in the delivery of compassionate nursing care.

## 2 Method

Three case study hospital sites were selected to explore how intentional rounding was implemented on the ground. Case study hospital sites were identified based upon the findings of a national survey conducted in Phase 2 of the study, to ensure they were as diverse as possible in their approach to intentional rounding. Hospital sites were purposively sampled to attain a range of geographically spread hospitals of varying sizes in a mixture of urban and rural locations. At each site, two case study wards (one acute, one care of older people) were identified by Trust staff to participate. The specialities of the participating acute wards were orthopaedic surgery, cardiology and respiratory medicine. Participating wards had a mixture of layouts, including nightingale wards, small bays and predominantly single rooms. Intentional rounding was carried out on these wards at either one or two-hourly intervals and researchers spent up to three weeks on each ward undertaking data collection.

Individual, semi-structured interviews were undertaken with 33 frontline nursing staff and 17 senior nurse managers/matrons/ward managers spread across the six case study wards (Table 1). Healthcare staff were purposively sampled, where possible, to attain a range of professions and grades and all staff participants were aware of and/or had personally undertaken intentional rounding. Thirty four patients and 28 family carers across the six case study wards also participated in semi-structured interviews whilst they were still receiving care in hospital. The recruitment of patients and family carers was guided by the nurse-in-charge of the ward, who advised researchers who was appropriate to take part (i.e. individuals who were particularly unwell or distressed were not approached). All participating patients had received intentional rounding whilst in hospital.

**Table 1:** Summary of interviewees.

Role of interview participant	Number of interviewees
Director of Nursing/Senior nurse manager/Matron	11
Ward manager	6
Senior staff nurse / junior ward sister (Band 6)	8
Staff nurse (Band 5)	13
Healthcare assistant (Bands 2 and 3)	12
Patients	34
Family carers	28

Interview schedules were specifically designed to elicit detailed reflections on the contexts, mechanisms and outcomes of intentional rounding and how it influenced the interviewee and those around them, as well as to obtain views on the purpose and value of intentional rounding (Interview schedules are reported in Harris et al., 2019). All participants gave informed consent and all interviews were audio-recorded and transcribed unless the interviewee requested otherwise. Transcripts were analysed using framework analysis (Ritchie and Spencer, 1994) to identify themes within the data and to test and refine the causal context-mechanism-outcome (CMO) configurations which were identified in the realist synthesis and used as part of the coding structure within the framework analysis (Harris et al., 2019).

### 2.1 Ethics

The study was approved by NHS Health Research Authority South East Coast - Surrey Research Ethics Committee (REC reference: 14/LO/1977) and all participants were informed that they were free to refuse to participate or withdraw from the study at any time.

## 3 Results

### 3.1 Views from the frontline nursing and managerial/senior nursing staff

All frontline nursing staff participants had personally undertaken intentional rounding and though none of the senior nursing managers had personally delivered it, they were all aware of how rounding was delivered and how they expected it to work. Both frontline nursing staff and senior nurse managers talked about the time constraints of nurses delivering care and how that perceived lack of time influenced the way intentional rounding was conducted. It was generally accepted that nurses would incorporate intentional rounding alongside other clinical or care activities and would be frequently interrupted when undertaking their rounds.

### 3.1.1 Intentional rounding does not improve standards of nursing care

Some participants focussed on the political origins of intentional rounding and expressed scepticism about the Government's motivations for introducing it, perceiving it to be a ‘knee jerk reaction’ in response to public disquiet:

*“… I'm aware this came out as a pre-emptive thing, didn't it, in light of the Francis Report, a pre-emptive strike by David Cameron to show that we're actually doing something after the horse had bolted, before it broke in the press”* (Staff nurse, Band 5).

*“… I mean, myself included, felt it was a little bit insulting, you know, that, actually, a piece of paper and this intention would fix* our *problem, when, actually, there's a lot more to the Francis report in relation to kind of staffing, the need and skill and resource.”* (Matron)

Others acknowledged the failures of nursing care and compassion highlighted in the Francis report and accepted there was a need to improve nursing care across hospital settings in general, in order to ensure patient safety. This was felt to be most important in hospitals that were providing poor care, with intentional rounding operating as a *‘standard-setter’* across these Trusts:

*“…. I suppose when it comes to things like the Francis report where people… had to drink out of plants and things like that… I suppose if something were in place to remind nursing staff to naturally give water, to naturally give the call bell, to naturally turn, to naturally make sure the bed's the lowest position possible. You know, if that's what's needed then I suppose it's needed”* (Staff nurse, Band 5)

*“…it's just making sure that patients aren't neglected and just sat there for hours, and no one's been to them to see if they're okay.”* (Staff nurse, Band 6).

However, most participants did not believe intentional rounding improved *their own* standard of nursing care or the care delivered by their Trust, as they rated both highly. Many believed intentional rounding was akin to what they were already doing and felt they would provide the same level of care with or without it:

*“… I don't think that it makes any difference in terms of how I care for my patients… before intentional rounding came in I was still going and seeing all my patients regularly, checking on all of these things, so… I just feel like it's a term that's been coined for something that we're already doing, they're just putting a name on it.”* (Senior staff nurse, Band 6)

Furthermore, for some senior nurse managers, there was concern that intentional rounding focussed only on the physical needs of patients and not their emotional or psychological needs. They worried that intentional rounding could reduce the scope of nursing care provided to patients towards a narrow focus on the fundamentals as well as the prevention of adverse events, leading to degradation of individualised care management:

*“… that's the minimum standard in a sense. I would rather have nurses who are able to articulate, ‘Mr Smith, I need to be* doing *this, this and this because I spotted that's what's going to make the difference for him while he's in hospital.’ … otherwise we have people sitting in hospital for ages because all we're doing is the fundamentals.”* (Executive Director of Nursing)

*“… can we add in something that reaches not just the physical needs, but… an opportunity to address some of the psychological and emotional needs of the patient which impact on their ultimate wellbeing and safety… how do we help staff to have that conversation, with an expectation that they should ask it..?”* (Senior Nurse Manager)

### 3.1.2 Intentional rounding improves the frequency – but not the quality – of nurse-patient interactions

A number of frontline nursing staff acknowledged that the process of undertaking intentional rounding increased the *frequency* of nurse communication with patients. They felt this increased frequency of communication was appreciated by patients and their carers, making patients feel *“cared for”* and *“valued”* and giving them confidence that they wouldn't be *“forgotten”* by nursing staff*.* One nurse referred to the increased frequency of communication as offering *“good customer service”* to patients and their carers, whilst a healthcare assistant believed it made patients, “*feel included”.*

Notably however, only three nursing staff and no senior nurse managers felt intentional rounding improved the *quality* of nurse-patient communication. Instead they felt it reduced nursing interaction to a list of tasks or closed questions to complete rather than giving nursing staff the opportunity to talk openly with patients:

***“…****I don't think it really does improve the quality because I think it is a kind of task that you're trying to do, you know, and get through, so I don't think that is improving the quality really.”* (Healthcare Assistant).

*“… the contact becomes transactional rather than enriching, so you're not having a conversation with that patient”* (Director of Nursing)

Others felt that intentional rounding could raise patients’ and carers’ expectations of how much time nursing staff should be spending with patients; an expectation that, in reality, nursing staff were not able to achieve.

### 3.1.3 Intentional rounding provides evidence that care has been provided but the accuracy of this evidence is unclear

It was clear that the greatest benefit of intentional rounding identified by nursing staff was that the associated documentation provided *“evidence”* or *“proof”* that nursing care had been delivered. The need to evidence the care one delivers seems to have been entrenched into the way of thinking of nursing staff, with many repeating a particular phrase, or words to that affect: ‘*if it's not documented, it's not done*’. That is not to say that nurses changed their actions because of intentional rounding: they said they were working as they always had done, but the intentional rounding documentation now gave them written proof of this:

***Interviewer:****“****Do you think if you didn't have to sign it, you might not go in [to a patient's room]?”***

*Staff Nurse:****“****Oh,* no*, I think I would go in but I think it's a good way of showing that I've gone in”* (Staff Nurse, Band 5).

The evidence generated by intentional rounding was useful to frontline nursing staff as it demonstrated care delivered, including where care delivery was delegated to Healthcare Assistants (HCAs), and provided them with what one participant called *‘psychological safety’* (i.e. making sure that they were doing the right things intentionally for their patients). A number of frontline nursing staff viewed intentional rounding as an important means of protecting oneself following an incident or complaint, suggesting that their testimony alone in such situations was insufficient:

*“… although people complain about paperwork, you do think if anything went wrong in a court of law, at least you've got something to back you up because your word isn't going to be everything.”* (Staff nurse, Band 5).

This highly structured and standardised evidence was also very useful to senior nurse managers, as it gave them assurance of a minimum standard of care delivered to all patients, particularly in unstable environments, which included shortages of staff, a high turnover and increased numbers of new or temporary staff. This evidence was found to be useful in dealing with untoward incident reporting, answering complaints and cases referred to the coroner:

*“I have been sat in meetings where we've had to talk to families before coronial processes and it's been a relief to the family and to me… to be able to take those charts and say “But this is the care that was provided to your loved one, this is the way in which we diligently cared for them, this is the way in which they interacted with the nurses on an hourly basis or a two hourly basis during the night”"* (Director of Nursing)

No one suggested that intentional rounding documentation offered any such protection to patients. Indeed, some frontline nursing staff and senior nurse managers voiced concerns about how accurately intentional rounding documentation reflected care delivered to patients and some admitted that the documentation was completed without rounding being undertaken. As a result, few nurse mangers relied upon intentional rounding documentation alone in response to a complaint but instead looked at the entire patient record:

“*…. it literally is a tick, tick, tick, tick, tick, tick, tick, tick, tick. Now, for me, that doesn't necessarily mean it was done*” (Matron)

*“.... sometimes* the *bottom part will be filled in, the pressure areas, every single hour and I just know that they haven't been checked every hour so they're just lying, it's just a lie” (*Staff nurse, Band 5).

### 3.1.4 Intentional rounding documentation does not increase compassion

Rather than improving standards or increasing individualised or compassionate care, there was suggestion that intentional rounding documentation could constrain it, with some participants stating that it increased frontline nursing staff workload and limited their time available to spend with patients:

*“… the task had become the documentation not the actual conversation or the care”* (Senior nurse manager)

*“… it takes me away from a patient rather than actually… getting me to go see my patients, it's actually keeping me away from them.”* (Staff nurse, Band 5).

Others noted the difference between ‘a good nurse’ and ‘a good documenter’ and felt frustrated when colleagues appeared to prioritise their documentation over their patients:

*“… I'm just like hitting my head going, 'the buzzer is going and you're sat here worried more about your documentation than actually answering your buzzer' … and I feel like sometimes people prioritise the documentation over actually seeing their patient”* (Staff nurse, Band 5)

Therefore, one nurse concluded that implementing intentional rounding was not an appropriate method to ensure compassionate patient care:

*“… there's no* documentation *that's going to go along and say 'hey, you should care more about your patients and check out if they've got water', you've either got that passion and you're already going to do that … and you've got that caring, compassionate nature that nurses have or you don't.”* (Staff nurse, Band 5).

### 3.2 Views from patients and carers

Patients and carers were generally satisfied with the amount and quality of nursing care received and were often eloquent and perceptive about the care that had been provided. However, it was difficult to determine whether, or to what extent, the care they described had taken place within an intentional rounding context. For example, almost all of the patients and carers interviewed commented upon the importance of positive communication, relationships and engagement with nursing staff, but it was rarely possible to link such comments directly to intentional rounding. This was because the majority of patients and carers had not been aware that intentional rounding was occurring: that is, only four patients and no carers said that intentional rounding had been explained to them. Of those who had not received an explanation, none expressed concern that they had not:

***Interviewer: “Okay. Would you have liked nursing staff to have explained that process to you..?”***

*Carer: “Not necessarily, because, you know, I think it's, you know, it's happening anyway, isn't it?”*

Instead, patients and carers talked more generally about the aspects of nursing care they valued most - the more relational aspects of communication - which made them feel cared for and reassured:

*“… to them you'*re not a patient, you are a person… I think that's the thing, you are a person, not a patient.”* (Patient)*

*“Well it just means that I can sleep at night, really. You know, that nothing horrible is going on, you know, that they listened to her, you know, and they don't fob her off which is good.”* (Carer)

The visibility of nursing staff was also valued by patients and carers, but again, their comments focussed on staff being visible on the ward in general rather than as part of a structured intentional round. Patients wanted to be able to get attention from nursing staff when they needed it, but were less concerned about the precise regularity or structure of rounding, so long as their needs were met and they felt they were being treated respectfully and with good humour. Indeed, some participants, when told by the interviewer of the existence of regular, scripted rounds, commented that they would not appreciate such an approach:

*“I don't think that'*s very people friendly really.”* (Carer)*

*“… I'd rather they had, they were allowed to use initiative and sensitivity, gut feeling, whatever you like to call it, you know, to the people because they get to know the people.”* (Patient)

Some participants, when asked whether they thought intentional rounding could help nursing staff anticipate and respond to their fundamental care needs, felt intentional rounding was better placed to address immediate care needs rather than anticipate future ones, such as a need to use the toilet or other aspects of comfort which might arise suddenly or unpredictably:

*“… they wouldn't necessarily come and ask if I want to go to the toilet but they would probably say “are you in pain?” They're more likely to concentrate on the medical side there and, you know, giving out pills and things, whereas going to the toilet, it's probably, would be more up to us to let them know”* (Patient)

## 4 Discussion

The findings of this study reveal, for the first time, the views of frontline nursing staff, senior nurse managers, patients and family carers on the role of intentional rounding in nursing. The findings suggest tensions between expectations – on the one hand that compassion in nursing is a core value and allows nurses to make sense of what they do, with the expectation to deliver intentional rounding in a mechanistic way, which provides utility in an increasingly audited and defensive risk culture. These themes are discussed here in relation to the findings.

Intentional rounding was implemented in England as a means of ensuring the comfort, safety and dignity of patients; a response to the ‘crisis of caring’ identified by the Francis report and an intervention to increase the delivery of compassionate nursing care. Yet, despite the good patient satisfaction, this study has found limited evidence that intentional rounding either increases standards of nursing care or improves nursing compassion. The systematised, structured approach to intentional rounding was seen by nursing staff and managers to emphasise transactional care delivery rather than relational, individualised patient care. Whilst some nursing professionals believed intentional rounding increased the frequency of nurse-patient communication and identified this as a means of providing good *‘customer service’*, few believed it improved the overall quality of these communications. Whilst increasing the frequency of nurse-patient communication might safeguard patients in settings where care delivery is poor, it is less apparent how useful this intervention would be in highly performing hospitals where this might actually reduce standards of care. Furthermore, whilst some nursing staff in this study felt that simply increasing the frequency of communication would be desirable to patients and their carers, the patients and carers interviewed said they valued the relational elements of their interactions with nursing staff; the humour, responsiveness and friendliness of communications which made them feel cared for and reassured. As the findings of this study show, such elements of communication are not a prominent feature in the intentional rounding protocol. Patients wanted to be able to get attention from nursing staff when they needed it and were less concerned about the precise regularity or structure of rounding nor convinced that it could help nurses anticipate their future needs. Indeed, dissatisfaction was expressed by some patients and carers towards the notion of a structured, scripted approach to nurse-patient communications and the need for a more *“people friendly”* approach was highlighted, which enabled nursing staff to use their *“initiative and sensitivity”.* Other research has also found it is the quality of interactions that patients value, in particular the importance of meaningful contacts and of feeling connected to nursing staff (Kenny, 2015; Maben et al., 2015). This demonstrates the tensions of intentional rounding around standardising, structured nursing input instead of accounting for the different needs and desires of patients.

Another tension highlighted in this study was that between an externally imposed performance management approach that focuses on what can be measured – for example, tasks, activities and episodes of care - and the more values-based relational care. Again, rather than increasing compassionate care, there was suggestion that intentional rounding could actually constrain it, with frontline nursing staff and senior nurse managers worrying that the main focus of intentional rounding was in completing the documentation rather than in the conversation with the patient. Differences were identified between a ‘*good nurse’* and a ‘*good documenter’*, and acknowledgement was made that no amount of documentation could make a nurse care more about their patient. These tensions, and the need for consistency in managing the relational aspects of providing patient care, have been previously identified (Kitson et al., 2014) but still remain a concern.

Documentation is an essential part of nursing; guidance from the Nursing and Midwifery Council (NMC) stresses the importance of clear and accurate record keeping (NMC, 2015;^2009^) and it is also a feature of organisational cultures that focus on managing risk. The recording of nursing documentation exists to protect the welfare of the patient, yet this study suggests intentional rounding documentation is primarily valued by nursing staff as a means of protecting themselves, by providing written evidence that they have provided care should incident or complaints arise. This appears to be due to what is perceived as the risk-averse and blame culture of the NHS healthcare environment and the prevailing attitude of, ‘*if it's not documented, it's not done*’. The need for such a safety net for nursing staff in wake of the Francis report is understandable, given the increased focus of media attention upon nursing and an organisational fear of “*being the next Mid Staffs”.* Intentional rounding can provide nursing with an important defence against allegations of poor practice at an individual practitioner, ward and organisational level. Whilst nursing staff may gain comfort from the protection this provides them, this study has shown that intentional rounding documentation does not always accurately reflect the care given. Such inaccurate documentation could undermine a nurse's credibility (Austin, 2010), provide a false sense of security to nursing managers and result in incorrect information being provided to family carers following incidents or complaints. This could lead to potential safety concerns for patients, as if inaccurate messages about the quality of patient care are being relayed to managers and family carers, neglectful or harmful care could be concealed. This also acts against healthcare professionals’ duty of candour with patients, colleagues, employers and organisations (General Medical Council and Nursing and Midwifery Council, 2015). Concerns about the accuracy of nursing documentation and the possible implications this may have are not new and have been highlighted elsewhere (Broderick and Coffey, 2013; Charalambous and Goldberg, 2016; Cheevakasemsook et al., 2006), but less is known about the particular concerns and issues associated specifically with intentional rounding documentation. The findings of this study suggest that healthcare managers should take into account the limitations of intentional rounding documentation as it reduces nursing activity to the measurement of tasks and the ticking of boxes. This results in oversimplifying at best, or at worst overlooking emotional support leading, in some cases, to unreliable reporting due to omission or commission.

In conclusion, these study findings question the usefulness of intentional rounding and whether it is the optimum intervention to support the delivery of compassionate nursing care to patients. Many different definitions of compassion have been highlighted in the nursing literature and due to its subjective nature, defining and understanding the true meaning of compassion remains complex. Indeed, compassion has been described as, *“a catch all word, a filler for the cracks which have been exposed in the care offered by the NHS as exemplified in the Francis Report”* (Jones and Pattison, 2016) and it is unclear how systematising nurse-patient interactions through the use of a structured tool such as intentional rounding might increase compassion. This study supports the notion that there is a danger of measuring what is easy to quantify in compassionate care, rather than what is important (Chochinov, 2007; MacLeod and McPherson, 2007). The structured approach of intentional rounding has been found to emphasise transactional care delivery and the emphasis on completing rounding documentation encourages staff to simply tick boxes on forms in order to protect themselves from complaint rather than deliver high quality, compassionate care. The question of whether or not quality nursing care is amenable to measurement is of great debate but it has been suggested that if governments impose the ‘appearance’ of compassion as a technique this reduces nurses to *“become merely an actor”* (Bradshaw, 2009). Indeed, one might question whether the intervention of intentional rounding, *“… asks nurses only to practice techniques such as the art of smiling, or the saying of warm words, in order that measures can be ticked and audited and data thereby gathered”* (Bradshaw, 2009).

To enable nurses to deliver compassionate care, appropriate support systems must be in place (Straughair, 2012b), and occupational stress and nurse burnout reduced, as these have been identified as key factors in the reduced occurrence of compassionate care (Firth-Cozens and Cornwell, 2009; Mills et al., 2015). Issues surrounding workforce turnover and nurse shortages also need to be addressed (Hayes et al., 2012; Roche et al., 2015). Introducing intentional rounding alone is not a sufficient means of increasing compassionate nursing care. The findings of this study suggest there is a need for international debate to consider whether or how far intentional rounding can achieve the nursing professions’ ambitions for the very best outcomes for patients. If an intervention to increase compassionate nursing care is required, it may be better to start afresh, rather than attempting to adapt the system that is currently implemented.

As we noted earlier in the paper, intentional rounding was announced by the government in the wake of the Francis Report that investigated a major health scandal. The intervention was abruptly imported from the US and with little evidence to support its relevance or suitability for the UK health system. The findings from this study show there is a trade-off between focusing on the transactional and the auditable activity of intentional rounding with relational nursing care that enacts compassion, kindness and empathy. It is not the first time that government policy for nursing has been announced without prior consultation or development with the profession. For example, in 1998 the UK Prime Minister Tony Blair made a surprise announcement to implement a new role of consultant nurse (Ross et al., 2011). It is in the nature of governments that policies can emerge with little warning. Therefore, it is our view that to prepare for this for the future, the nursing profession needs to become more confident about drawing on evidence to challenge announcements which have not been the subject of consultation, but which have a direct impact on the delivery of nursing care*.*

### 4.1 Limitations of the study

Although geographically spread, case study sites were limited to England only and not the rest of the UK. Case study sites were selected using the responses to the national survey conducted in phase 2 to provide breadth of context within which intentional rounding was implemented. However, being able to achieve the depth of data required for detailed examination of intentional rounding constrained the number of case study sites that could be included. This may limit the generalisability of findings. Furthermore, Trust nursing managers selected the wards to be included in the research and this may have introduced bias. Finally, it was often difficult to determine whether, or to what extent, the care patients and family carers described within their qualitative interviews had taken place within an intentional round. This was because the majority of patients and carers had not been aware that intentional rounding was occurring.

## 5 Conclusion

This large-scale, theoretically-driven study of intentional rounding – the first of its kind – demonstrates that intentional rounding does not improve the delivery of compassionate nursing care and therefore does not address the policy imperative it was intended to target. Intentional rounding documentation is primarily valued by nursing staff as a means of protecting themselves rather than patients; by providing written proof or ‘evidence’ that they have provided care should incident or complaint arise. The systematised approach of intentional rounding emphasises transactional care delivery rather than relational, individualised patient care and has the potential to commodify nursing and reduce the scope of nursing care to a minimum standard, leading only to a narrow focus on the fundamentals as well as the prevention of adverse events. As Albert Einstein famously said, “Not everything than can be counted counts, and not everything that counts can be counted”. It is our view that there is a need for a wider and international debate within the nursing profession to consider what its desired outcomes are and whether intentional rounding is the best way of achieving them.

For more information about the study, please visit the project website at: https://www.journalslibrary.nihr.ac.uk/programmes/hsdr/130787/#/summary-of-research

## CRediT authorship contribution statement

**Sarah Sims:** Conceptualization, Data curation, Formal analysis, Writing - original draft, Writing - review & editing. **Mary Leamy:** Data curation, Formal analysis, Writing - original draft, Writing - review & editing. **Ros Levenson:** Conceptualization, Formal analysis, Writing - original draft, Writing - review & editing. **Sally Brearley:** Conceptualization, Formal analysis, Writing - original draft, Writing - review & editing. **Fiona Ross:** Conceptualization, Formal analysis, Writing - original draft, Writing - review & editing. **Ruth Harris:** Conceptualization, Data curation, Formal analysis, Writing - original draft, Writing - review & editing.
